# AI‐Optimized Vanadium Oxide Multilayers for More Than 20‐fold Enhancement in Bolometric Performance

**DOI:** 10.1002/advs.202514344

**Published:** 2026-01-28

**Authors:** Jin‐Hyun Choi, Hyoung‐Taek Lee, Jeonghoon Kim, Miju Park, Seungjoon Sun, Gye‐Hyeon Kim, Myung‐Ho Kwon, Hyeong‐Ryeol Park, Changhee Sohn

**Affiliations:** ^1^ Department of Physics Ulsan National Institute of Science & Technology Ulsan Republic of Korea; ^2^ Pohang Accelerator Laboratory POSTECH Pohang Gyeongbuk Republic of Korea; ^3^ i3system Company Daejeon Republic of Korea

**Keywords:** machine learning, metal‐insulator transition, microbolometers, phase‐transition materials, vanadium oxides

## Abstract

Phase‐transition materials such as vanadium dioxide (VO_2_) inherently exhibit non‐linear and hysteretic behavior, which limits their applicability in devices like infrared bolometric sensors that require linear and non‐hysteretic responses. To circumvent this issue, nonstoichiometric VO_x_ has been widely used in infrared bolometers despite its degraded phase transitions and resultant lower temperature coefficient of resistance (TCR) compared to stoichiometric VO_2_. Achieving both a high TCR and a linear, non‐hysteretic response has therefore remained a major bottleneck in advancing microbolometer technology. In this study, we present a multilayer approach using machine‐learning‐optimized W_x_V_1‐x_O_y_ thin films with varying doping ratios to address these challenges. By stacking layers with different W doping levels and employing genetic algorithm optimization, we achieve tailored linear/flat TCR profiles and significantly reduced hysteresis. These multilayer systems simultaneously achieve a high TCR and low electrical noise even under complementary metal‐oxide semiconductor (CMOS)‐compatible growth conditions, resulting in a universal bolometric performance 23.6 times greater than that of commercial materials. This work demonstrates a general methodology for achieving both a large and linear response to external stimuli, which can be widely adopted not only for microbolometers but also for other technologies.

## Graphical abstract

Machine‐learning‐optimized W_x_V_1‐x_O_y_ multilayer thin films with graded doping achieve a high TCR (7.3 % K^−^^1^), reduced hysteresis, and low noise under CMOS‐compatible growth conditions. This approach overcomes the long‐standing trade‐off in microbolometers between linear response and performance, offering a universal bolometric parameter greatly enhanced over commercial materials, with broad applicability to sensing technologies.

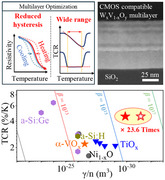

## 1 Introduction

Phase transitions, as collective phenomena of many‐body systems, play vital roles in numerous technologies; however, their inherently non‐linear and hysteretic nature often limits their applicability in certain systems [1, 2, 3]. The linear response of physical properties is critical in myriad technologies, as non‐linear responses cause signal distortion and design complexity, making them less suitable for systems requiring consistent performance [4, 5, 6]. Notable examples include capacitive materials like (Hf, Zr)O_2_ in dynamic random‐access memory (DRAM) and bolometric materials like Si, TiO_x,_ or VO_x_ in infrared sensing applications. These material responses—such as charge accumulation or changes in resistivity—must maintain a linear relationship with the applied stimuli, such as electric voltage or temperature changes induced by infrared light [7, 8]. In contrast, materials undergoing phase transitions, including metal‐insulator transitions (MIT), ferroelectricity, and superconductivity, exhibit inherently large response, but non‐linear and often hysteretic responses to external stimuli [9, 10, 11]. While large responses, in principle, enable transformative advances—such as significantly enhanced integration densities for DRAM or improved sensitivity in sensing technologies—their practical implementation remains challenging [2, 12, 13]. The key barrier of applying these materials to device applications lies in leveraging between the disadvantages of their non‐linearity and hysteresis and the advantages of their large responses [12, 14].

The prototypical example of such obstacles can be found in infrared bolometric sensing applications by utilizing the MIT in VO_2_ [1, 15]. A bolometer operates by detecting infrared light through materials with a high temperature coefficient of resistance (TCR), where the absorption of infrared radiation induces a temperature change, consequently altering the material's resistance. VO_2_ and its related compounds have garnered substantial attention for such applications due to their first‐order MIT near room temperature, which inherently provides a large TCR. However, the TCR (response) of pure VO_2_ not only exhibits extreme non‐linearity and hysteresis with respect to temperature changes (external stimuli) but also remains only in a narrow temperature window, rendering it impractical for real‐world applications. While nonstoichiometric amorphous VO_x_ compounds have been employed in microbolometers to reduce non‐linearity and hysteresis, this approach comes at the cost of significantly diminishing the TCR, limiting its maximum value to approximately 2 % K^−1^ and being a major bottleneck to further enhance the sensitivity of microbolometers [16, 17].

To overcome the intrinsic non‐linearity, hysteresis, and narrow transition range of VO_2_, multilayer architectures have recently been proposed as a strategy to achieve more linear and thermally stable responses [18, 19]. As one of the pioneering efforts, Émond et al. [18] demonstrated a W_x_V_1‐x_O_2_ multilayer (50/50/50 nm with *x* = 0–0.025) deposited on LaAlO_3_ (100) at ∼550 °C, achieving a TCR of 10.4 % K^−1^ with minimal variation. This pioneering study showed that stacking can mitigate the intrinsic limitations of VO_2_, yet its high growth temperature and use of LaAlO_3_ substrates make it incompatible with complementary metal‐oxide semiconductor (CMOS) fabrication, restricting its applicability to practical device platforms. More recently, Wheeler et al. [19] performed numerical optimization of an ALD‐derived ten‐layer W:VO_2_ structure using material parameters measured experimentally, predicting a nearly constant TCR (≈6.7 ± 0.9 % K^−1^) over a 50 K temperature range. Although providing valuable theoretical insight, the multilayer structure was not experimentally realized, and its large number of layers and potential dopant interdiffusion would challenge reproducible fabrication. Collectively, these studies established the conceptual potential of multilayer architectures to enhance VO_2_ functionality, yet it remains experimentally unverified whether such improvements can be achieved under CMOS‐compatible conditions and lead to tangible advances in device‐level performance.

**FIGURE 1:**
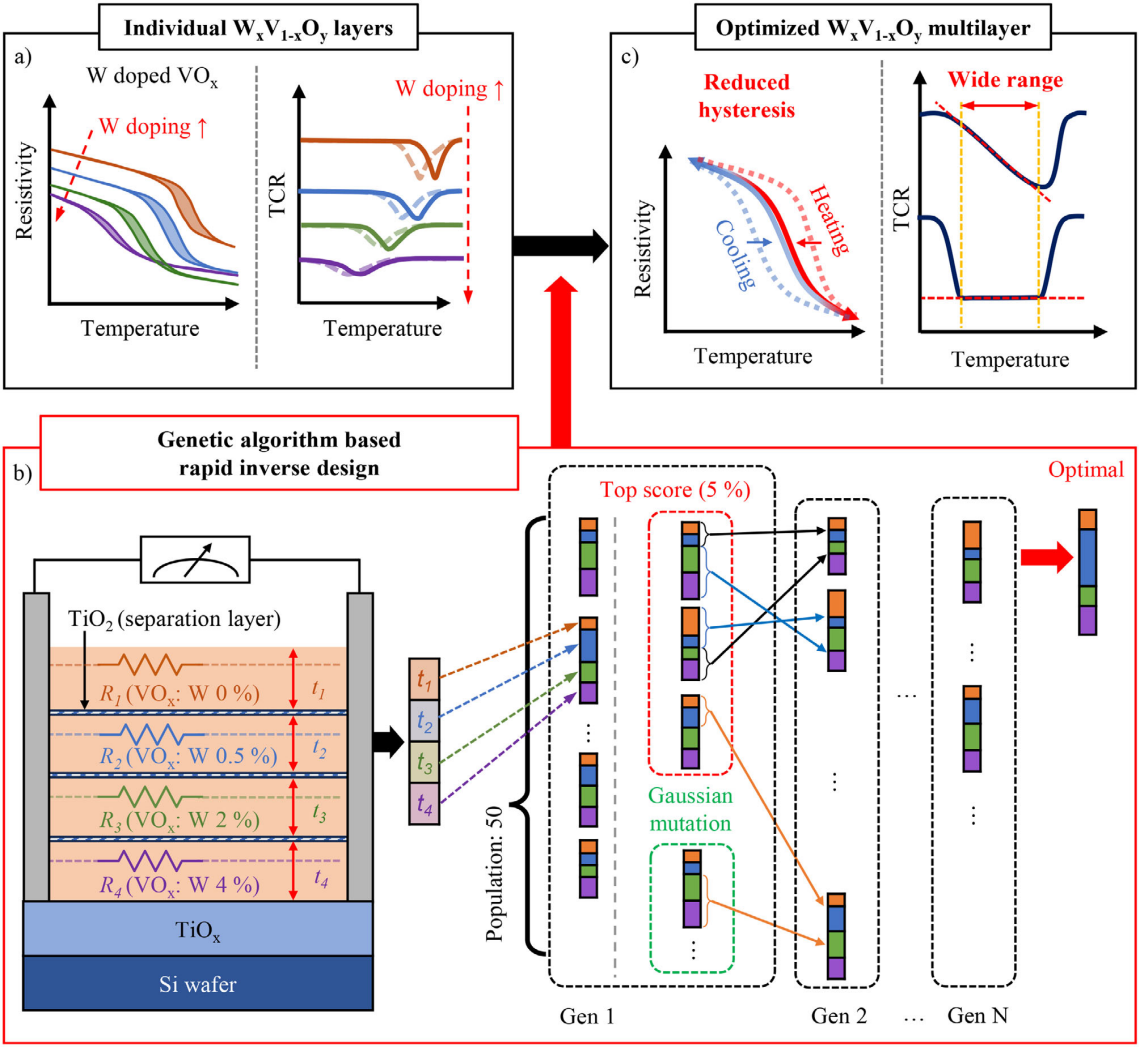
Schematics of machine‐learning‐based inverse design for optimizing W_x_V_1‐x_O_y_ multilayer structures with tailored bolometric properties. (a) Schematics of the evolution of resistivity and TCR versus temperature with an increasing W doping ratio in W_x_V_1‐x_O_y_ thin films. The solid and dashed lines in TCR curves correspond to the curves obtained with increasing and decreasing temperatures, respectively. (b) Left: The schematics of W_x_V_1‐x_O_y_ multilayer structures with four different doping levels (x = 0, 0.005, 0.02, and 0.04). To prevent intermixing of W dopants, 4 nm TiO_2_ layers are inserted between the different doping layers. The total resistance is modeled by treating the multilayers as a series of parallel resistors. To meet the parallel resistor model, resistances are measured with a side electric contact. Right: A genetic algorithm with Gaussian mutation is employed to optimize W_x_V_1‐x_O_y_ multilayer structures. The total resistance of multilayer structures is evaluated by varying the thickness of each layer (*t*_1_, *t*_2_, *t*_3_, and *t*_4_). The fitness functions are carefully designed to achieve constantly high (CH) or linearly high (LH) TCR curves with a wide operation temperature range and reduced hysteresis, which is required for infrared microbolometer applications. Details of the algorithm are provided in Note S1. (c) Targeted resistivity and TCR curves of the optimized W_x_V_1‐x_O_y_ multilayer via genetic algorithm are shown.

Building on these earlier efforts, we present an experimentally validated, computationally guided, and CMOS‐compatible design of VO_x_ multilayers that achieve a linear, high‐TCR, and less‐hysteretic bolometric response. As schematically shown in Figure 1a, W doping in VO_x_ can sensitively modulate its critical temperature and overall MIT behaviors. If we stack VO_x_ layers with different W doping (Figure 1b), the global resistivity of the heterostructure will be described as a series of parallel resistors [20]. Although the global resistivity of heterostructures can be estimated using a simplified model, designing structures with optimal bolometric performance is far from intuitive. This is because multiple objectives must be simultaneously satisfied —achieving a large TCR near room temperature, maintaining a constant or linear TCR over a broad temperature range, and minimizing hysteresis. To address this multi‐objective optimization problem, we employed a genetic algorithm (GA)–based machine learning framework to determine the optimal thickness ratio of each layer. Guided by the machine‐learning approach, we synthesized optimized W doped VO_x_ heterostructures under CMOS‐compatible conditions (Figure 1c). We found the expected linear/non‐hysteretic responses of our heterostructure as well as its superior performance. The optimized heterostructure exhibits a high TCR of 7 % K^−1^ and low 1/*f* noise with Hooge parameters of *γ*/*n* of 6.90 × 10^−32^ m^3^, resulting in 23.6 times greater universal parameter of bolometer than commercially available sensing materials.

## 2 Results and Discussion

We employed a GA incorporating Gaussian mutation to optimize the thickness ratios of W_x_V_1‐x_O_y_ multilayers for bolometric applications. GA, an optimization method inspired by natural selection and genetic evolution, is particularly effective for solving multilayer optimization problems [21, 22, 23]. As illustrated in Figure 1b, the multilayer models consist of four W_x_V_1‐x_O_y_ layers with varying tungsten doping levels of 0, 0.5, 2, and 4 %. The thicknesses of each layer (*t*_1_, *t*_2_, *t*_3_, and *t*_4_) are treated as genes, forming a chromosome. The resistivity of each layer, which will be used to simulate the total sheet resistance of multilayers, is separately measured with a physical property measurement system (PPMS) and presented in Figure S1. Starting with randomly generated fifty sets of chromosomes in the first generation, the algorithm selects the most “fit” chromosomes as parents based on specific fitness functions, as explained in supplementary note 1. These functions assign higher scores to chromosomes exhibiting (1) a large TCR near room temperature, (2) a constant or linear TCR over a broad temperature range, and (3) reduced hysteresis. The top 5 % of chromosomes based on these scores are selected as parents to produce offspring through crossover, which combines genetic information from two parents, and mutation, which introduces random alterations to the genetic code. Further details are provided in the Methods section and supplementary materials.

**FIGURE 2:**
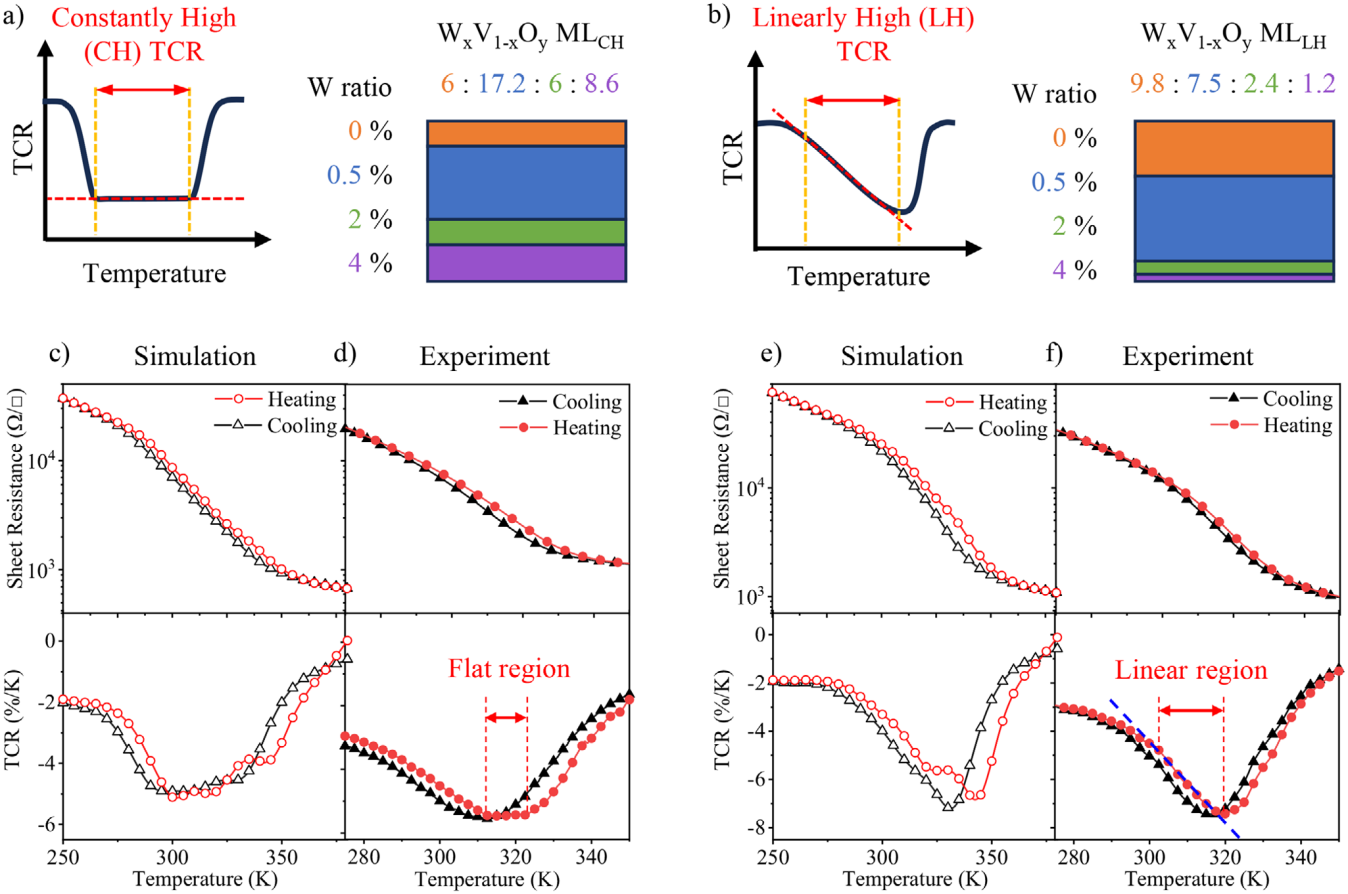
Optimized W_x_V_1‐x_O_y_ multilayers for constantly high (CH) and linearly high (LH) TCR curves. Schematic representations of TCR curves and optimized multilayer structures for (a) CH and (b) LH optimizations. The optimal thickness ratios for W_x_V_1‐x_O_y_ multilayers with W concentrations of 0, 0.5, 2, and 4 % obtained from CH and LH optimizations are also presented. (c) Simulated and (d) experimental sheet resistance/TCR curves for the CH optimized multilayer. Both the simulation and experimental TCR curves show a constant TCR of ∼5.5 % K^−1^ near room temperature. (e) Simulated and (f) experimental sheet resistance/TCR curves for the LH optimized multilayer. A linear TCR from 4.5 % K^−1^ to 7.3 % K^−1^ is observed within a 20 K temperature window (a blue dashed line). These results highlight the effective modulation of TCR and hysteresis through multilayer design.

Building on this, our GA approach effectively identifies optimized multilayer structures with customizable TCR curves tailored for bolometric applications. As mentioned earlier, it is essential for the TCR curves of bolometric materials to remain constant or at least linear, without inflection points, across a wide temperature range. To meet these requirements, we explored two configurations: constantly high (CH) TCR and linearly high (LH) TCR, achieved by modifying the fitness functions (supplemental note 1). The optimal thickness ratios for the four layers are depicted in Figure 2a,b, while the corresponding simulated TCR curves, shown in Figure 2c,e, exhibit the desired constant and linear behaviors within ≈20 K temperature windows. These results validate that, with appropriately designed fitness functions, the GA approach provides an effective method for configuring TCR curves, as further detailed in Figure S2. Remarkably, despite the total number of possible thickness combinations being about 1.3 million cases, the GA efficiently identified optimal solutions by sampling only near 0.28 % (converged at 74th gen) of the total search space, highlighting its exceptional efficiency.

To demonstrate our approach, we synthesized optimized VO_x_ and W‐doped VO_x_ heterostructures on Si wafers with precision. Undoped V_2_O_5_ and W‐doped V_2_O_5_ targets were used in pulsed‐laser deposition (PLD). Since the accuracy of the optimization heavily depends on the input data, the thicknesses and sheet resistances of W‐doped VO_x_ single layers (with W doping ratios of 0, 0.5, 2, and 4 %) deposited on TiO_x_‐buffered Si substrates were carefully measured using X‐ray reflectometry (XRR) (Figure S3) and PPMS (Figure S1), respectively. The thicknesses of the optimized W_x_V_1‐x_O_y_ multilayers were further analyzed by scanning transmission electron microscopy with energy dispersive X‐ray spectroscopy (STEM‐EDS) and XRR. Both STEM‐EDS (Figure S4) and XRR (Figure S5) confirm that the experimentally fabricated CH‐optimized thin films follow the optimal thickness ratios, with deviations of approximately 4.8 % per layer.

Another key point we wish to emphasize is that our heterostructures were grown under CMOS‐compatible conditions, which are crucial for practical device applications. Notably, most previous studies reporting a TCR higher than that of commercialized materials employed both high growth temperatures exceeding 500 °C and non‐silicon substrates [1, 18, 24, 25, 26]. However, such high synthesis temperatures exceed the 350 °C limit of the sacrificial layer (typically polyimide) [27], which is used to achieve thermal isolation of bolometric materials during microbolometer fabrication, thereby rendering these materials impractical for real‐world applications. In this study, on the other hand, we synthesized VO_x_ multilayer on commercial TiO_x_‐based microbolometer wafers provided by i3system [28] with a low growth temperature of 300°C (Figures S6 and S7). The strict restriction in growth temperature and utilization of commercial microbolometer wafer signify high feasibility of our approach for practical applications.

The optimal multilayer samples exhibit significantly reduced hysteresis while maintaining flat and linear TCR profiles, consistent with the simulations. Figure 2d,f shows the experimental TCR curves of the optimized multilayer samples for CH and LH TCR configurations, respectively. For the CH TCR multilayer, a nearly constant TCR value of ≈5.4 % K^−1^ with an extremely low standard deviation (0.026 % K^−1^) is achieved with a response temperature range of 12.3 K. In the case of the LH TCR multilayer, the TCR spans from 4.7 to 7.4 % K^−1^ within temperature windows of 17.2 K. Hysteresis, measured as center‐to‐center temperature differences for CH and peak‐to‐peak differences for LH, is reduced to 5.3 K for CH and 3.1 K for LH. Both multilayer samples demonstrate remarkably reduced hysteresis, despite the individual layers exhibiting large hysteresis behaviors (11.4 K for pure VO_x_ thin film), as shown in Figure S1. Experimental results align qualitatively with the simulations for both configurations, validating the effectiveness of our approach. Minor quantitative differences between simulations and experiments likely arise from thickness errors in individual layers and interfacial effects between W_x_V_1‐x_O_y_ layers and the TiO_2_ separation layer (Figure S8), which is introduced to prevent migration of dopants.

**FIGURE 3:**
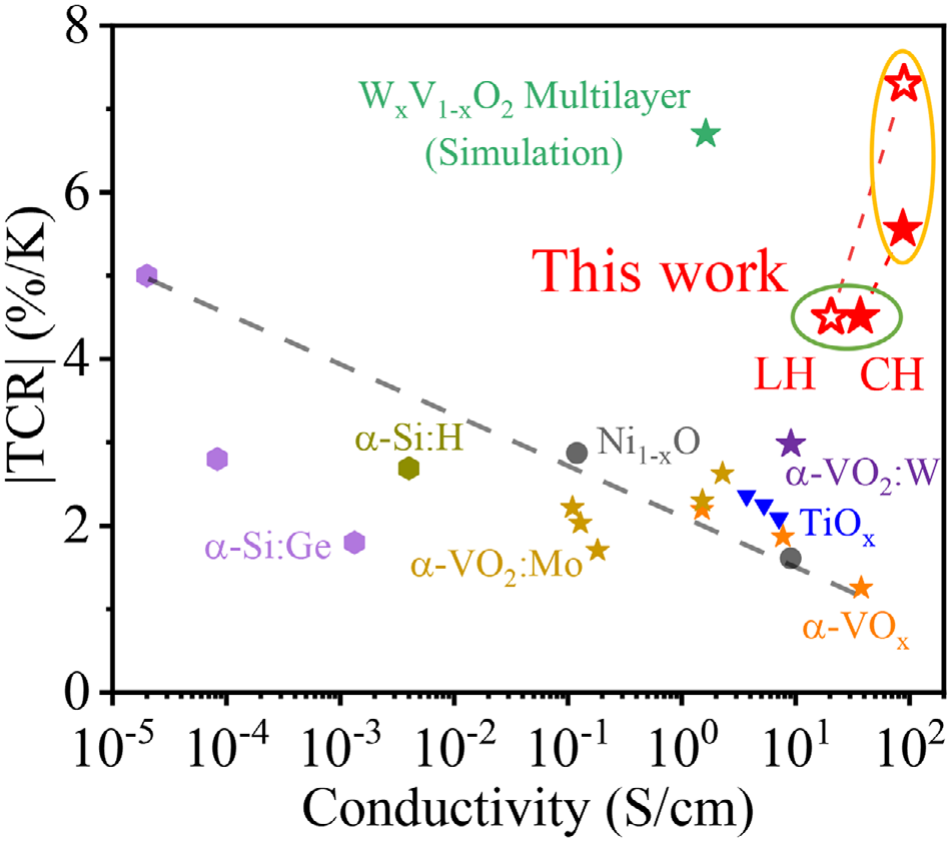
Comparison of TCR versus conductivity for W_x_V_1‐x_O_y_ multilayers and other CMOS‐compatible bolometric materials. The TCR and conductivity of CH (solid red stars) and LH (hollow red stars) W_x_V_1‐x_O_y_ multilayers are obtained at 300 K (green circle) and 320 K (yellow circle). The data are compared with other CMOS‐compatible bolometric materials, including α‐Si:Ge, Ni_1–x_O, α‐VO_x_ (1.3 < x < 2.0), and others (see Table S1). In general, high TCR and high conductivity are considered as a tradeoff, as indicated by the grey dashed line. Both CH and LH W_x_V_1‐x_O_y_ multilayers exhibit high TCR and high conductivity at both 300 K and 320 K, demonstrating their potential for high‐performance bolometric applications.

Another notable strength of our heterostructure approach is the simultaneous achievement of both high TCR and high conductivity. For bolometer device applications, achieving both high TCR and high conductivity is critical, with the latter directly influencing the noise level of the voltage signal. However, these two properties are inherently antagonistic to each other. Figure 3 illustrates the relationship between TCR and conductivity for known CMOS‐compatible bolometric materials [28, 29, 30, 31, 32, 33, 34]. As highlighted by a grey dashed line, there is a clear inverse relationship between TCR and conductivity. In materials with low conductivity, such as semiconductors and insulators, carrier density exhibits exponential temperature dependence, typically leading to a large TCR. Conversely, in metals with high conductivity, the carrier density remains nearly constant with temperature, and only the mean free path of carriers changes with polynomial dependence on temperature, resulting in low TCR. The inherent difficulty in achieving both high TCR and high conductivity is evident in previous studies on Si‐based bolometers [35], where increasing carrier density effectively reduced noise levels but simultaneously diminished TCR due to this tradeoff.

In contrast, our W_x_V_1‐x_O_y_ multilayers exhibit both high conductivity and high TCR, demonstrating exceptional performance compared to other bolometric materials [28, 29, 30, 31, 32, 33]. Despite their large TCR observed in Figure 2d,f, CH and LH multilayer exhibits a high conductivity of a few tens of S cm^−1^ in the temperature range from 300 to 320 K. As highlighted by the solid (CH) and hollow (LH) stars with respect to 300 K (green circle) and 320 K (yellow circle) in Figure 3, our heterostructures deviate from the typical inverse relationship between TCR and conductivity in both room temperature and high temperature (320 K). Our results show similar behavior to that of the multilayer simulations proposed by Wheeler et al. [19], thereby experimentally demonstrating the effectiveness of the multilayer approaches for simultaneously achieving both high conductivity and large TCR. This unique behavior arises because our method utilizes the large, non‐linear MIT occurring in one specific layer at a given temperature, while the other layers remain metallic, preserving high conductivity across a wide temperature range.

**FIGURE 4:**
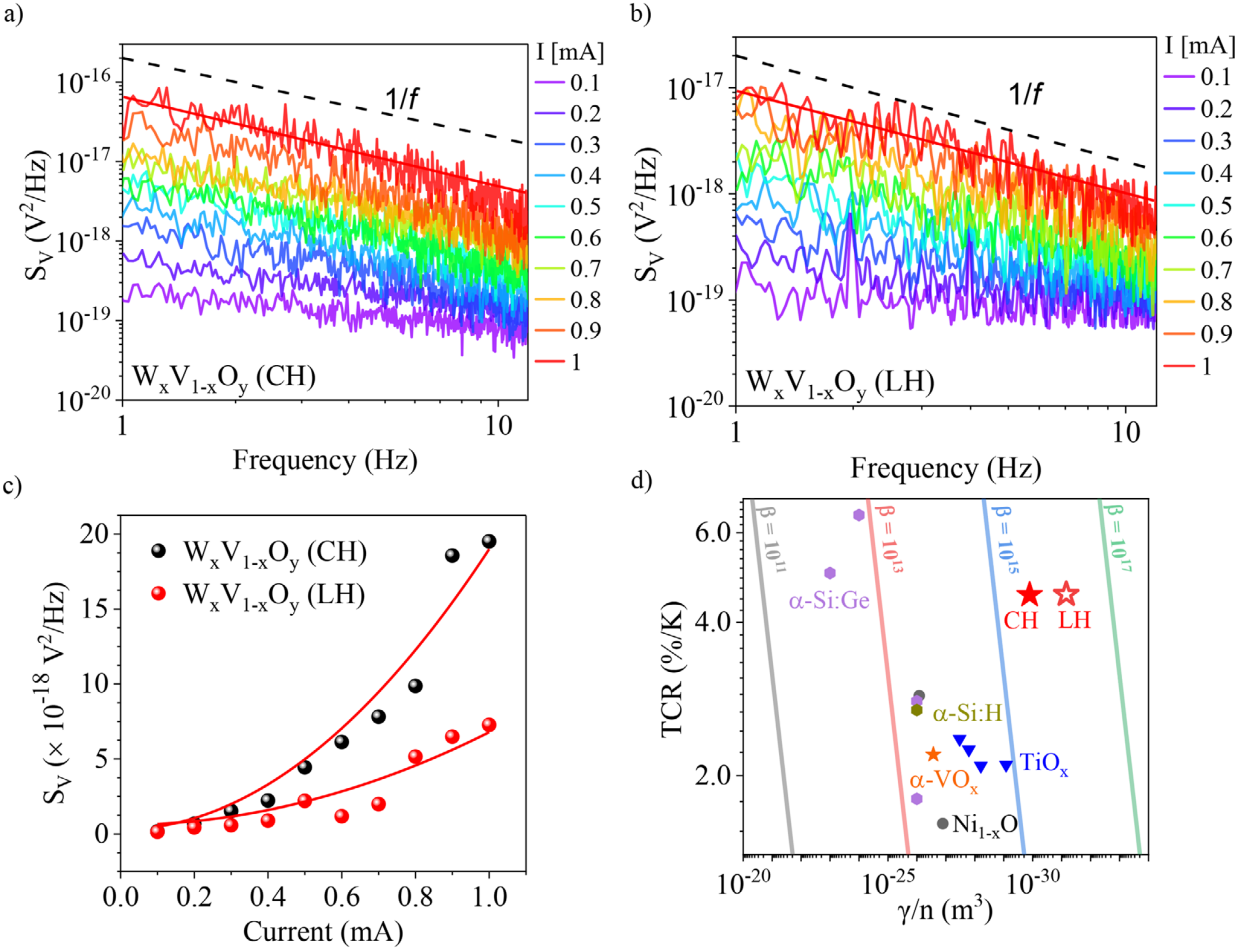
Noise characteristics and universal parameter of bolometer (*β*) of W_x_V_1‐x_O_y_ multilayers with LH and CH configurations. (a,b) Electrical noise spectral densities of the CH and LH multilayers measured using a spectrum analyzer. The red lines represent 1/*f* fittings of the noise spectra under a 1 mA current bias, and the black dashed lines indicate ideal 1/*f* reference slopes. (c) Noise voltage spectral density (*S_V_*) at 1 Hz for CH and LH multilayers under varying current biases. The bolometric performance parameter (*γ*/*n*) is extracted by fitting the *S_V_* at 1 Hz using the fitting function “A·*I* ^2^+B” (red curves). (d) Comparison of TCR versus *γ*/*n* for W_x_V_1‐x_O_y_ multilayers and other bolometric materials (Table S1). To evaluate the general performances of bolometric materials, the universal parameter of bolometer (*β*) can be defined as |TCR|/√(*γ*/*n*). The *β* of W_x_V_1‐x_O_y_ shows a 5.5 times higher figure of merit for the CH configuration and 23.6 times for the LH configuration compared to commercial bolometric materials. The black, red, blue, and green lines correspond to reference *β* benchmark values of 10^11^, 10^13^, 10^15^, and 10^17^, respectively, highlighting the superior performance of W_x_V_1‐x_O_y_ multilayers.

Due to their high conductivity, W_x_V_1‐x_O_y_ multilayers exhibit exceptionally low 1/*f* noise compared to conventional bolometric materials. Figure 4a,b presents the noise spectral densities of W_x_V_1‐x_O_y_ multilayers with CH and LH configurations at room temperature (300 K), under current bias ranging from 0.1 to 1 mA in 0.1 mA of increments. Both configurations clearly show 1/*f* noise behavior. In bolometric applications, the empirical Hooge's relation [36] is widely used as a benchmark for noise performance, expressed as:

(1) $$S_{V}=\frac{\gamma }{n}\times \frac{R^{2}I^{2}}{\Omega \times f^{\alpha }}$$

where *S_V_* is the voltage noise spectral density, *γ* is Hooge's parameter, *n* is carrier density, *R* is the resistance of the samples, *I* is the current applied to the samples, *Ω* is the volume of the sample, *α* is a dimension parameter, and *f* is the measuring frequency. In equation (1), *S_V_* should scale quadratically with the applied current, as confirmed in Figure 4c. Based on this relation, the Hooge's parameter per carrier density (*γ*/*n*), a key metric for noise‐performance, can be extracted. The calculated *γ*/*n* values are 1.27 × 10^−30^ m^3^ for CH and 6.90 × 10^−32^ m^3^ for LH, both of which are lower values than those of other bolometric materials, including VO_2_ [37, 38]. This reduction in noise can be attributed to the increased carrier density resulting from W doping, which is known to enhance carrier density by more than two orders of magnitude [39, 40]. In undoped VO_2_, the *γ*/*n* values typically show around an order of 10^−30^ m^3^, but can fluctuate by 2 to even more than 4 orders of magnitude due to the complex nature of its MIT [37, 38]. Given that our W_x_V_1‐x_O_y_ multilayers incorporate W‐doped VO_x_ layers, it is reasonable to observe *γ*/*n* values as low as 10^−32^ m^3^, owing to the enhanced carrier density. To further validate the reliability of our noise measurements, we also measured the noise spectra of TiO_x_ thin films (Figure S9e), which yielded *γ*/*n* value of 7.87 × 10^−30^ m^3^, consistent with a previous report [28]. These results demonstrate that W_x_V_1‐x_O_y_ multilayers achieve lower *γ*/*n* compared to commercial bolometric materials—by a factor of 6.5 for CH and 120.3 for LH configuration—surpassing even TiO_x_, which, to the best of our knowledge, represents one of the lowest values among commercial bolometric materials.

Assessed relative to other bolometric materials, the W_x_V_1–x_O_2_ multilayers demonstrated a universal parameter of bolometer (*β*) over 23.6 times higher than other CMOS‐compatible materials [28, 29, 32, 33, 41], highlighting their superior performance. The *β* is commonly used to evaluate bolometric performance [36] by accounting for both TCR and *γ*/*n*, and it is defined as:

(2) $$\beta =\frac{\left|TCR\right|}{\sqrt{\gamma /n}}$$

Figure 4c shows TCR and *γ*/*n* values of various bolometric materials, alongside reference *β* benchmarks (solid lines). To the best of our knowledge, the highest *β* in commercially available bolometric materials is the order of 10^15^ [28]. In contrast, the LH (CH) multilayer W_x_V_1–x_O_2_ heterostructures exhibit a *β* more than 23.6 times (5.5 times) larger than that of commercial TiO_x_. This remarkable enhancement highlights the potential of the multilayer approach not only to boost sensitivity but also to significantly reduce noise levels, offering a novel pathway toward the development of ultrahigh‐performance microbolometers.

## 3 Conclusion

The experimental results demonstrate that W_x_V_1‐x_O_y_ multilayers, optimized through machine‐learning‐based inverse design, successfully achieve both high TCR and a linear, reduced hysteretic response, with a nearly 20‐fold higher bolometric performance. Machine‐learning‐based optimization methods are inherently adaptable, allowing for the incorporation of additional variables to enhance material performance further [42, 43]. We expect that, by introducing greater complexity, such as different dopants, deliberate modulation of oxygen vacancies, and consideration of interfacial effects, the approach can be expanded to achieve even higher performance in the future.

Furthermore, our W_x_V_1‐x_O_y_ heterostructures were synthesized with careful consideration for CMOS and Readout Integrated Circuit compatibility. All samples were grown on commercial microbolometer wafers at a low temperature of 300°C to align with the maximum thermal tolerance of bolometer device structures. Although PLD was used in our experiments, the materials employed, such as W‐doped VO_x_ and TiO_2_, are well‐suited for sputtering techniques, enabling the feasibility of large‐area growth on 8‐inch wafers for future applications. The operational temperature range of our multilayer heterostructure (15–20 K) is narrower than that of conventional microbolometers; however, for highly sensitive bolometric sensors requiring high TCR, this range is sufficient and can be effectively managed using Peltier devices. We believe this approach holds significant promise for the development of high‐performance microbolometers, particularly for military applications, and demonstrates clear potential for real‐world implementations.

Finally, we believe that our multilayer approach, combined with machine learning optimization, has the potential to be broadly applied across various fields. For instance, perovskite structures ATiO_3_ (A = Ba, Sr, Ca) demonstrate significantly higher dielectric constants compared to conventional capacitive materials [44]. A similar multilayer approach using ATiO_3_ could achieve a large dielectric constant while maintaining a linear and reduced hysteretic response to external voltage. Furthermore, advancing modern technology often requires maximizing the figure of merit of materials, a task complicated by the inherently antagonistic nature of their physical properties. Examples include balancing TCR and conductivity in microbolometers [45], dielectric constant and bandgap in capacitors [46], and the Seebeck coefficient with electrical and thermal conductivity in thermoelectric [47]. As demonstrated in this study, the integration of multilayer structures with machine learning optimization provides a promising pathway to overcome these trade‐offs and enhance material performance.

## 4 Experimental Methods

### 4.1 Sample Preparation

VO_x_ thin films are synthesized on TiO_x_/SiO_2_/Si wafers (i3system) by pulsed‐laser deposition. Un‐doped V_2_O_5_ target (Toshima Manufacturing Co., ltd) and Various W doped V_2_O_5_ targets are used in pulsed‐laser deposition. W doped V_2_O_5_ targets were synthesized by the solid‐state reaction method with mixing WO_3_ powder (99.9 %, Sigma–Aldrich Corp.) and V_2_O_5_ powder (99.6 %, Sigma–Aldrich Corp.) with respect to the atomic ratio, which were sintered at 600°C for 6 h. The TiO_2_ targets were synthesized by sintering TiO_2_ powder (99.9 %, Sigma–Aldrich Corp.) at 600°C for 6 h. The growth conditions for thin films are the substrate temperature T = 300°C, oxygen partial pressure P = 10 mTorr, laser fluence 1.5 J cm^−2^, and repetition frequency 5 Hz. The TiO_2_ separation layers are grown between VO_x_ layers with an oxygen partial pressure as 13 mTorr. The distance between the target and substrate was set at 50 mm. The cooling process was performed under the same grown oxygen partial pressure after the deposition was completed.

### 4.2 Thickness and Structural Characterization of Thin Films

The thicknesses of W doped VO_x_ single layers are measured by X‐ray reflectometry (XRR) (D8 advance high‐resolution X‐ray diffractometer, Brucker). The XRR measurements are conducted with Cu *K‐α*1 wavelength, scan angle ranges from 0.3 to 5 ° with 0.01 ° increment and 0.01 ° s^−1^ of scan speed. The thickness of the CH W_x_V_1‐x_O_y_ multilayer sample was also measured by XRR with the 3A Hard X‐ray Scattering Beamline in PLS‐II of the Pohang Accelerator Laboratory. The XRR data were fitted with multilayer models (GenX) [48]. The cross‐sectional images were obtained from CH samples dissected by focused ion beam (FIB) (Helios 450HP FIB, FEI) with high‐resolution transmission electron microscopy (HR‐TEM) (JEM‐2100F, JEOL). With an energy dispersive X‐ray spectroscopy module attached to HR‐TEM, cross‐sectional compound mapping images were also obtained, which show dispersion of O, Si, Ti, and V atoms. The cross‐sectional images of VO_x_ thin film deposited on a silicon wafer with/without TiOx buffer layers were obtained by TEM and their fast Fourier transform.

### 4.3 Electrical Characterizations

The resistivities for machine‐learning optimizations are obtained with 20 nm‐thick W_x_V_1‐x_O_y_ thin films grown on TiO_x_‐buffered Si wafers by a physical property measurement system (PPMS, Cryogenic Co., Ltd.). All measurements were conducted with van der Pauw methods with symmetric 5 × 5 mm^2^ square samples, where sheet resistance could be obtained by multiplying π/ln2 to measured resistances. To ensure that measurements follow the parallel resistor approaches, four corners of square samples were slightly scratched and applied Ag epoxy (H20E, Epoxy Technology, inc.), wired with Au wire to make good electronic contacts. GE varnish (CMR‐direct Co.ltd) was applied to achieve thermal contact between the samples and the PPMS probe.

To evaluate the bolometric performance of our samples, noise spectral densities were measured for W_x_V_1‐x_O_y_ multilayer samples and TiO_x_ (as a reference sample). To reduce contact issues during noise measurements, Au thin film was deposited on each sample by DC sputtering with a stencil mask. As shown in Figure S10, the distance between the contact pads was approximately 770 µm with 5 mm of width. A load resistor with a resistance at least 100 times greater than that of the sample was connected in series. A Keithley 2450 sourcemeter was used to apply bias current to the samples. The linear *I*–*V* responses with respect to the bias current were confirmed for each sample to ensure the measurements are conducted in an ohmic regime. The electrical noise was amplified using a preamplifier (SR570), and the noise spectral densities were measured with an FFT analyzer (SR770) [49].

### 4.4 Genetic Algorithm Optimizations

The genetic algorithm‐based inverse design was conducted using the optimization toolbox in MATLAB (The MathWorks Inc.). Genetic algorithms, a global optimization technique, do not rely on exploring a single solution but instead maintain a population of candidate solutions, effectively addressing the issue of becoming trapped in local optima. The chromosomes for the genetic algorithm were composed of thickness genes, which were regulated to have values of 0–200 Å, and the total thickness was restricted to have 200 Å. The scores for each chromosome are evaluated by *f*_CH_ or *f*_LH_, which are described in Note S1. The maximum iterations were set as 200 generations, and in each iteration, the top 5 % genes were selected as parents for the next generation. The mutation rate decreases over successive generations following a Gaussian mutation algorithm [50].

## Author Contributions

J.‐H.C., H.‐T.L., and C.S. conceptualized this work. M.‐H.K and i3system Inc. provided substrates and consulted with CMOS compatibility. J.‐H.C. synthesized and characterized the thin films. H.‐T.L. designed fitness functions and conducted machine‐learning optimizations. J.‐H.C., S.S., and M.P. performed electric sheet resistance measurements. J.‐H.C. and J.K. conducted the noise spectra measurements. G.‐H.K conducted XRR measurement. J.‐H.C., H.‐T.L., J.K and C.S. analyzed the experimental data. J.‐H.C., H.‐R.P., and C.S. wrote the paper with input from all coauthors.

## Conflicts of Interest

The authors declare no conflict of interest.

## Supporting information




**Supporting File**: advs74066‐sup‐0001‐SuppMat.docx.

## Data Availability

The data that support the findings of this study are available from the corresponding author upon reasonable request.
