# Supplementary material for: AI‐Optimized Vanadium Oxide Multilayers for More Than 20‐fold Enhancement in Bolometric Performance
Source: Adv Sci (Weinh). 2026 Jan 28;13(19):e14344. doi: 10.1002/advs.202514344 (PMC13045228; doi:10.1002/advs.202514344)
Supplement: Supplementary file 1 — Supporting File: advs74066‐sup‐0001‐SuppMat.docx. [file ADVS-13-e14344-s001.docx]

Supporting Information

**AI-optimized vanadium oxide multilayers for more than 20-fold enhancement in bolometric performance**

Jin-Hyun Choi^1†^, Hyoung-Taek Lee^1, 2†^, Jeonghoon Kim^1^, Miju Park^1^, Seungjoon Sun^1^, Gye-Hyeon Kim^1^, Myung-Ho Kwon^3^, Hyeong-Ryeol Park^1*^, and Changhee Sohn^1*^

^1^Department of Physics, Ulsan National Institute of Science & Technology, UNIST-gil 50, Ulsan, 44919, Republic of Korea.

^2^Pohang Accelerator Laboratory, POSTECH, 80, Jigokro-127-beongil, Nam-gu, Pohang, Gyeongbuk 37673, Republic of Korea.

^3^i3system Company, Techno 5-ro 69, Daejeon, 34014, Republic of Korea.

**Contents**

**Supplementary Note 1.** Design principles of fitness functions in genetic algorithm (GA) optimization

**Supplementary Note 2.** Electronic characteristics of W_x_V_1-x_O_y_ single layers

**Supplementary Note 3.** TCR curves of high scoring chromosome obtained from GA

**Supplementary Note 4.** Growth rates measurements of W doped V_2_O_5_ targets with XRR

**Supplementary Note 5.** Structure of W_x_V_1-x_O_y_ multilayer with CH configuration

**Supplementary Note 6.** Achieving CMOS compatibility with low temperature growth

**Supplementary Note 7.** CH W_x_V_1-x_O_y_ multilayer without TiO_2_ separation layer

**Supplementary Note 8.** The *I*-*V* curve and quadratic response of noise spectral densities of W_x_V_1-x_O_y_ multilayers and TiO_x_ film

**Supplementary Note 9.** Images of electric contact pads deposited on samples for noise spectrum measurements

**Supplementary Note 10.** Bolometric performances of previously reported materials

**References.**

**Supplementary Note 1. Design principles of fitness functions in genetic algorithm (GA) optimization**

The fitness functions for optimizing the TCR curves of W_x_V_1-x_O_y_ multilayer are carefully designed to obtain constantly high (CH) TCR and linearly high (LH) TCR response. The sheet resistance of the multilayer is considered as a series of parallel resistors as described in Equation S1,

$\frac{1}{R_{Total}}=\sum_{i=1}^{4} \frac{t_{i}}{\rho_{i}}$ (S1)

, where *R*_Total_ is a total sheet resistance of multilayer, *t*_i_ is the thickness of each layers, and *ρ*_i_ resistivity of each layer. Using the calculated *R*_Total_, the TCR of *R*_Total_ can be evaluated using the Equation S2.

${TCR}_{Total}\left( R_{Total}, T \right)=\frac{d(\ln\left( R_{Total} \right))}{dT}$ (S2)

For convenience, *R*_Total_ and *TCR*_Total_ would be described as simply *R* and TCR in describing following fitness functions. Using Equation S1 and Equation S2, fitness functions are designed to evaluate high scores for non-hysteretic linear TCR curves. Firstly, the fitness function *f*_CH_ is designed to achieve non-hysteretic and CH TCR response (Figure 2(a)),

$f_{CH}\left( R \right)=\int_{280}^{360} [\frac{|TCR(T)|}{max(\left| TCR \right|)}-\omega\left( R_{H}\left( T \right)-R_{C}\left( T \right) \right)]dT$ (S3)

, where *ω* represents weight parameter to modulate the influences of hysteresis. The hysteresis is considered as (*R*_H_ – *R*_C_), which *R*_H_ and *R_C_* represent resistance in heating curve and cooling curve, respectively. The first term of *f*_CH_ is designed to achieve maximizing area of TCR curves under desired temperature range, which is normalized by maximum value of TCR, giving more scores to square-like flat TCR curve shape. Combining area maximizing term and reducing hysteresis term, *f*_CH_ evaluates constantly high and non-hysteretic curves more highly throughout the progression of generation in GA. Secondly, *f*_LH_ is designed to achieve non-hysteretic and LH TCR response (Figure 2(b)). Secondly, the fitness function *f*_LH_ for achieving non-hysteretic and LH TCR response (Figure 2(b)) is designed as following,

$f_{LH}\left( R \right)=\int_{280}^{T_{x}} [\left| TCR\left( T \right) \right|-\omega\left( R_{H}(T)-R_{C}(T) \right)-\omega'\left( \frac{d^{2}|TCR(T)|}{dT^{2}} \right)]dT$ (S4)

, which *ω* is a weight parameter of hysteresis, *ω’* is a weight parameter of second derivative of TCR and *T_x_* is an inflection point which considers a tolerance *α*. The inflection point, *T_x_* is defined as a temperature point where the second derivative of TCR exceeds the value of *α*, which is set as 0.2. The first term of Equation S4 consists of the integration of TCR, which is introduced to achieve high TCR by maximizing area. To consider the broadness of high TCR region, the integration range varies depends on *T_x_*, which gives more scores where *T_x_* is away from starting point (280 K). By doing this, both high and linear TCR over a broad temperature range can be achieved. The hysteresis is considered the same as in Equation S3, which is described in second term in Equation S4. Finally, we incorporate a penalty based on the second derivative of the TCR curves to enforce linear TCR curve. The procedural optimization results for top scoring cases are illustrated in Figure S3, which clearly shows how GA can effectively propagate to its desired optimal TCR profiles.

**Supplementary Note 2. Electronic characteristics of W_x_V_1-x_O_y_** **single layers**


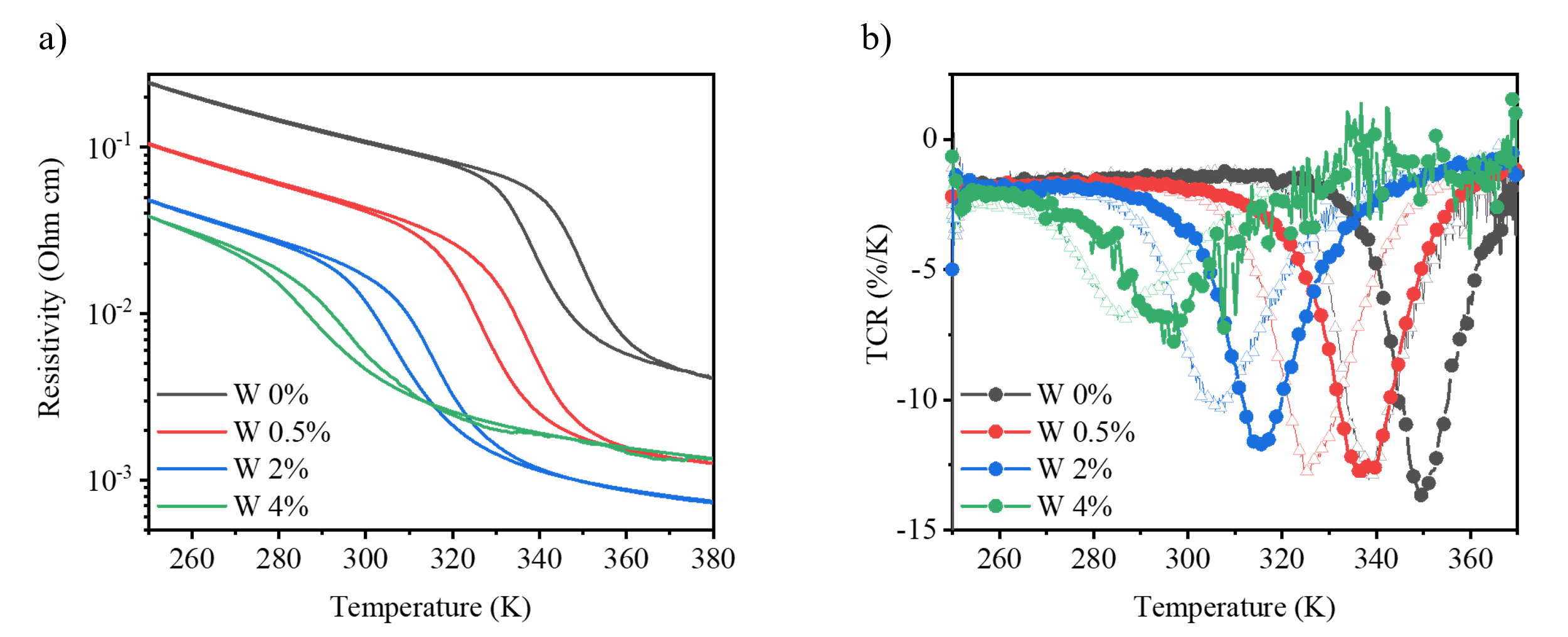


**Figure S1**. (a) Resistivity and (b) TCR *vs*. temperature curves of W_x_V_1-x_O_y_ thin films with different W doping ratios.

**Supplementary Note 3. TCR curves of high scoring chromosome obtained from GA**


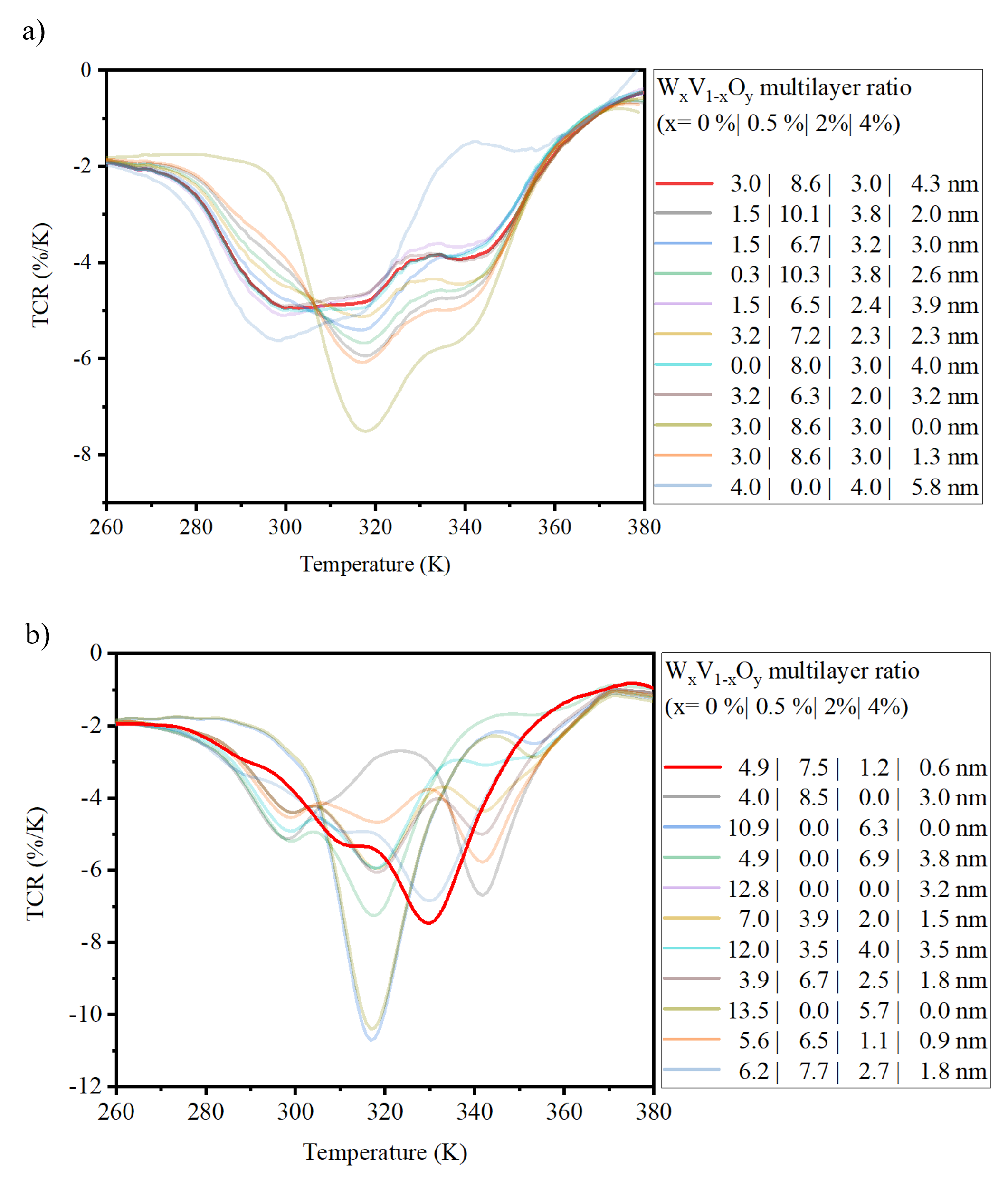


**Figure S2**. Machine-learning optimized TCR curves obtained using (a) CH fitness function and (b) LH fitness function. The top-scoring cases are higlighted with red lines.

**Supplementary Note 4. Growth rates measurements of W doped V_2_O_5_ targets with XRR**


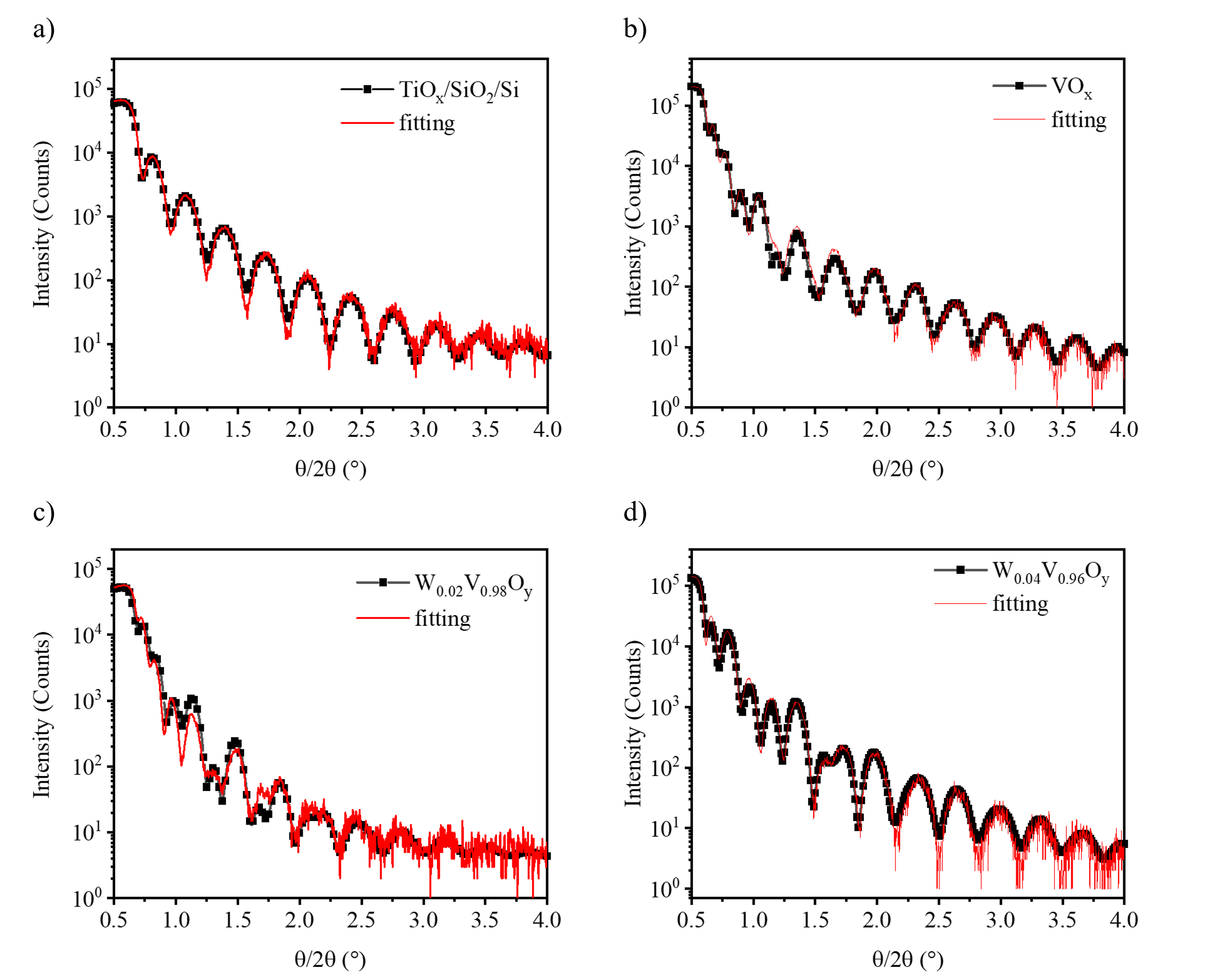


**Figure S3**. XRR measurements of (a) TiO_x_-buffered substrates and W-doped VO_x_ films deposited onto buffered-substrates with doping ratios of (b) 0 %, (c) 2 % and (d) 4 %. By fitting the XRR data (red lines) with multilayer models (GenX) ^[1]^, we obtain the precise thickness and growth rate for each W doped VO_x_ layer.

**Supplementary Note 5. Structure of W_x_V_1-x_O_y_** **multilayer with CH configuration**


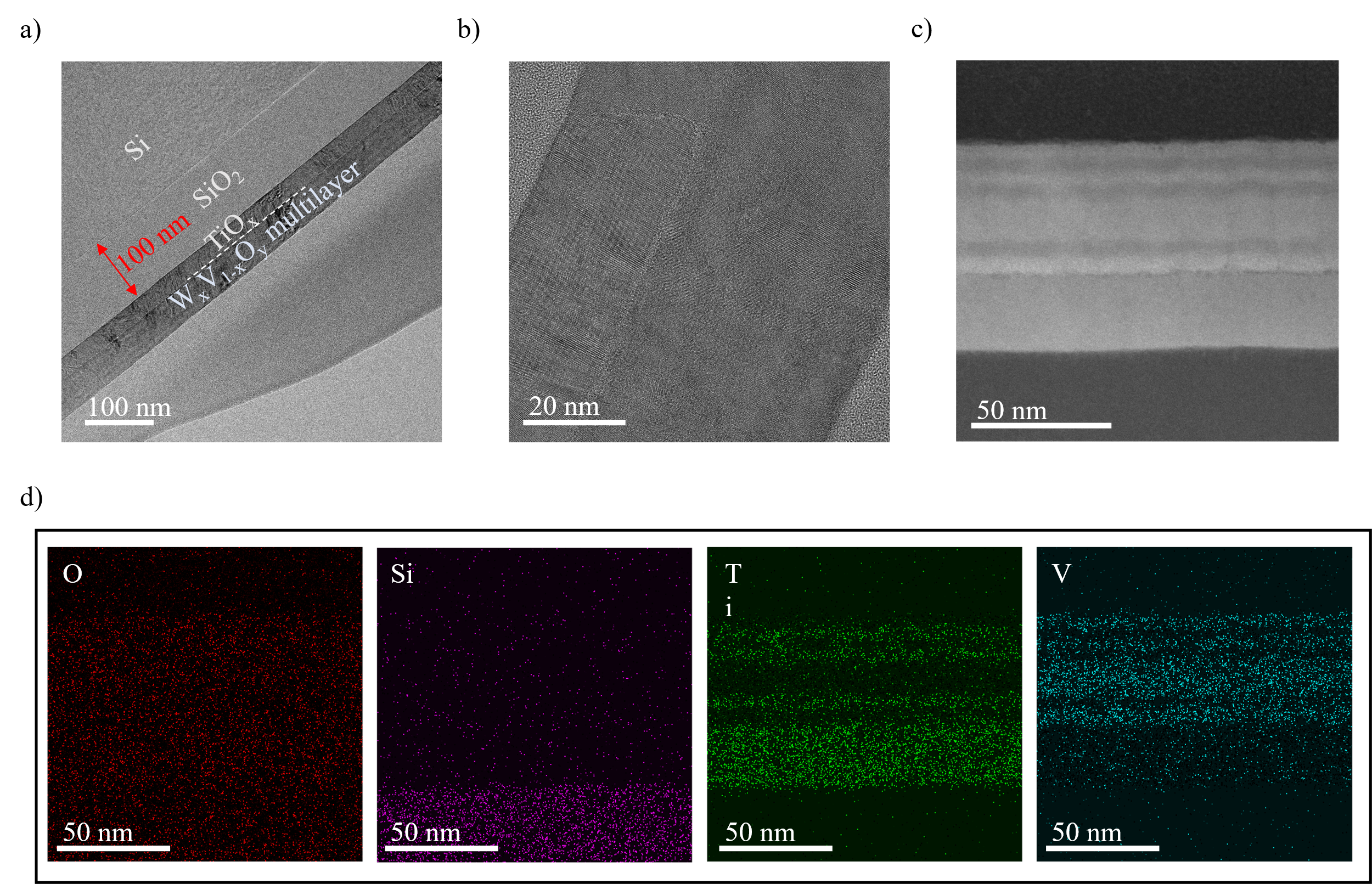


**Figure S4**. (a-c) Cross-sectional STEM images of CH W_x_V_1-x_O_y_ multilayer. (d) EDS elemental mapping images obtained from STEM image for O, Si, Ti, and V.


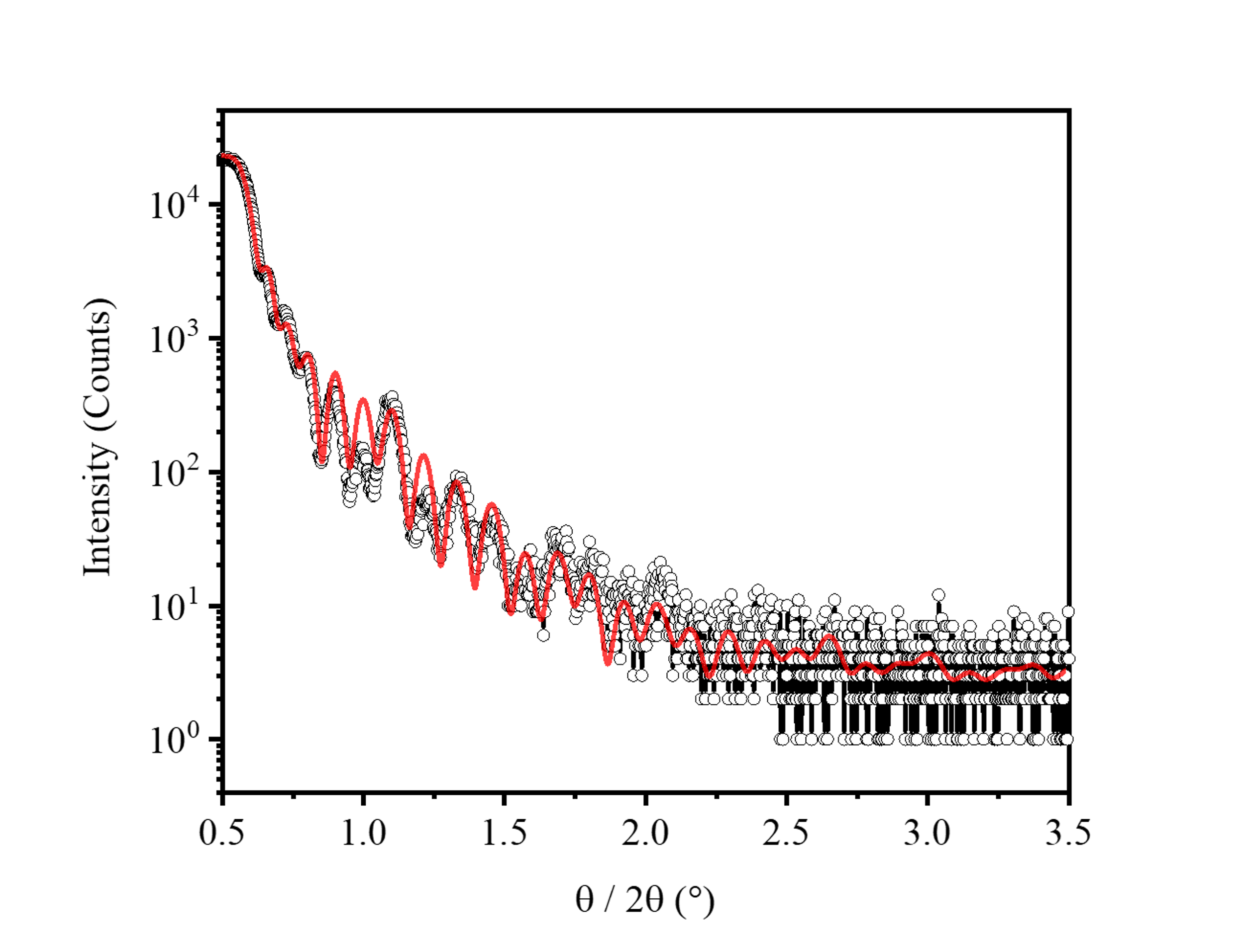


**Figure S5**. XRR measurement of the CH W_x_V_1-x_O_y_ multilayer.

The multilayer of CH configuration was measured using STEM. As shown in Fig. S4(a) and (b), the thicknesses of SiO_2_ and TiO_x_ buffer layer are determined to be 100 nm and 25 nm, respectively. The multilayer composition is shown in Figure S4(c), which clearly shows four well-defined layers of W doped VO_x_ thin films, separated by TiO_2_ layer. The composition of CH multilayer was further analyzed by EDS elemental mapping images, shown in Figure S4(d). The EDS mapping images indicate Ti atoms are positioned in buffer layer and separation layers, while V atoms are positioned in the multilayer with desired thicknesses.

Figure S5 exhibits the XRR data (open circles) of W_x_V_1-x_O_y_ multilayer obtained by 3A Hard X-ray Scattering Beamline in PLS-II and the best fitting results (red solid line). The fitted line confirms that our multilayer configuration is well-matched with desired thicknesses, with only ~5 % variation per layer between the expected and measured thicknesses.

**Supplementary Note 6. Achieving CMOS compatibility with low temperature growth**


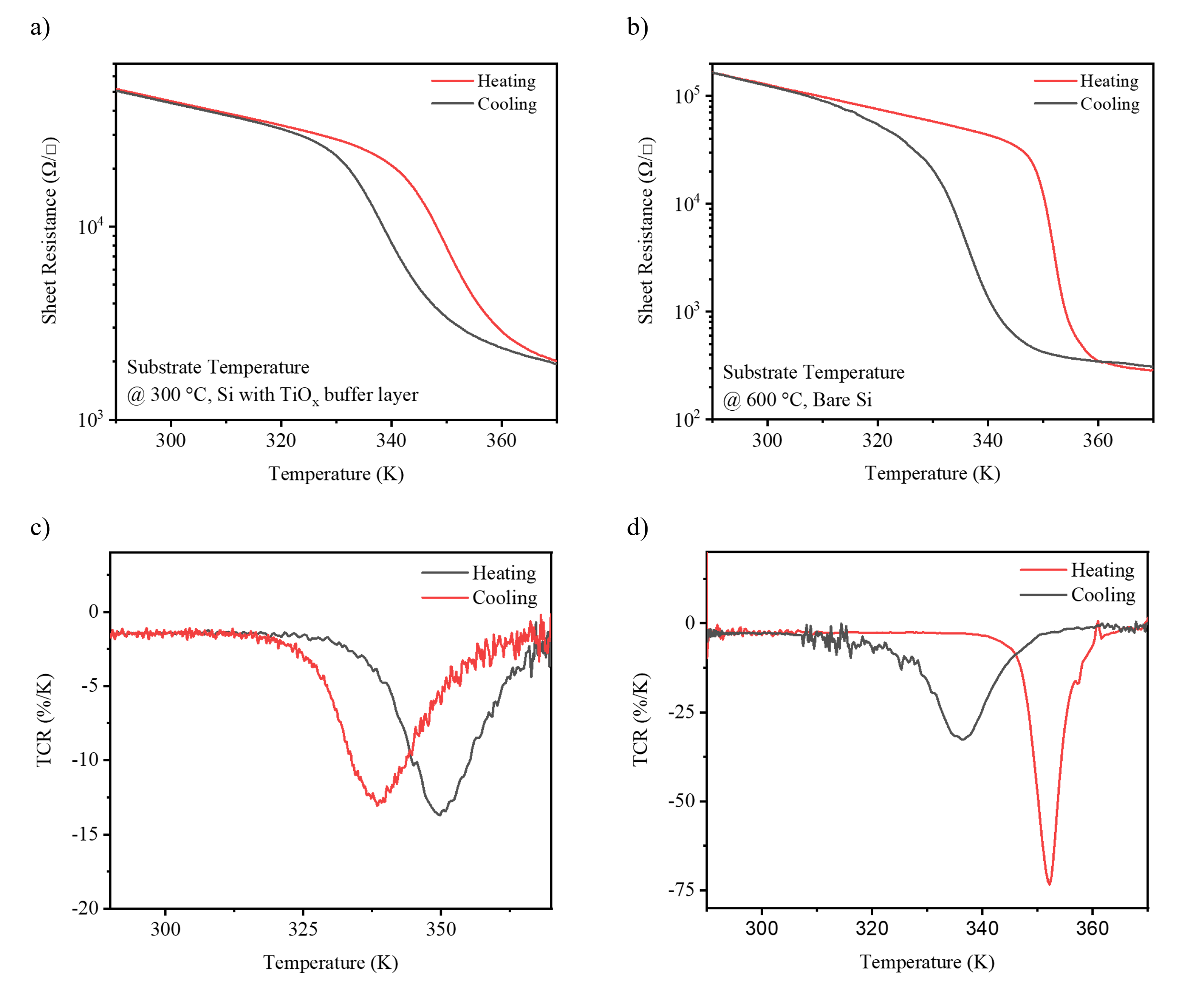


**Figure S6**. Sheet resistance *vs*. temperature curves of the VO_x_ thin film deposited on (a) a Si wafer with TiO_x_-buffer layer at 300°C, and (b) a Si wafer at 600 °C. TCR curves of (c) a Si wafer and (d) a TiO_x_-buffered Si wafer are obtained from the resistance *vs*. temperature data above. Despite the low growth temperature, the VO_x_ thin film grown on TiO_x_-buffered Si wafers exhibits clear metal-insulator transition behavior. Note that, the VO_x_ film directly grown on Si wafer at 300 °C show a resistance higher than 1 MΩ, incapable to providing reliable resistivity data.


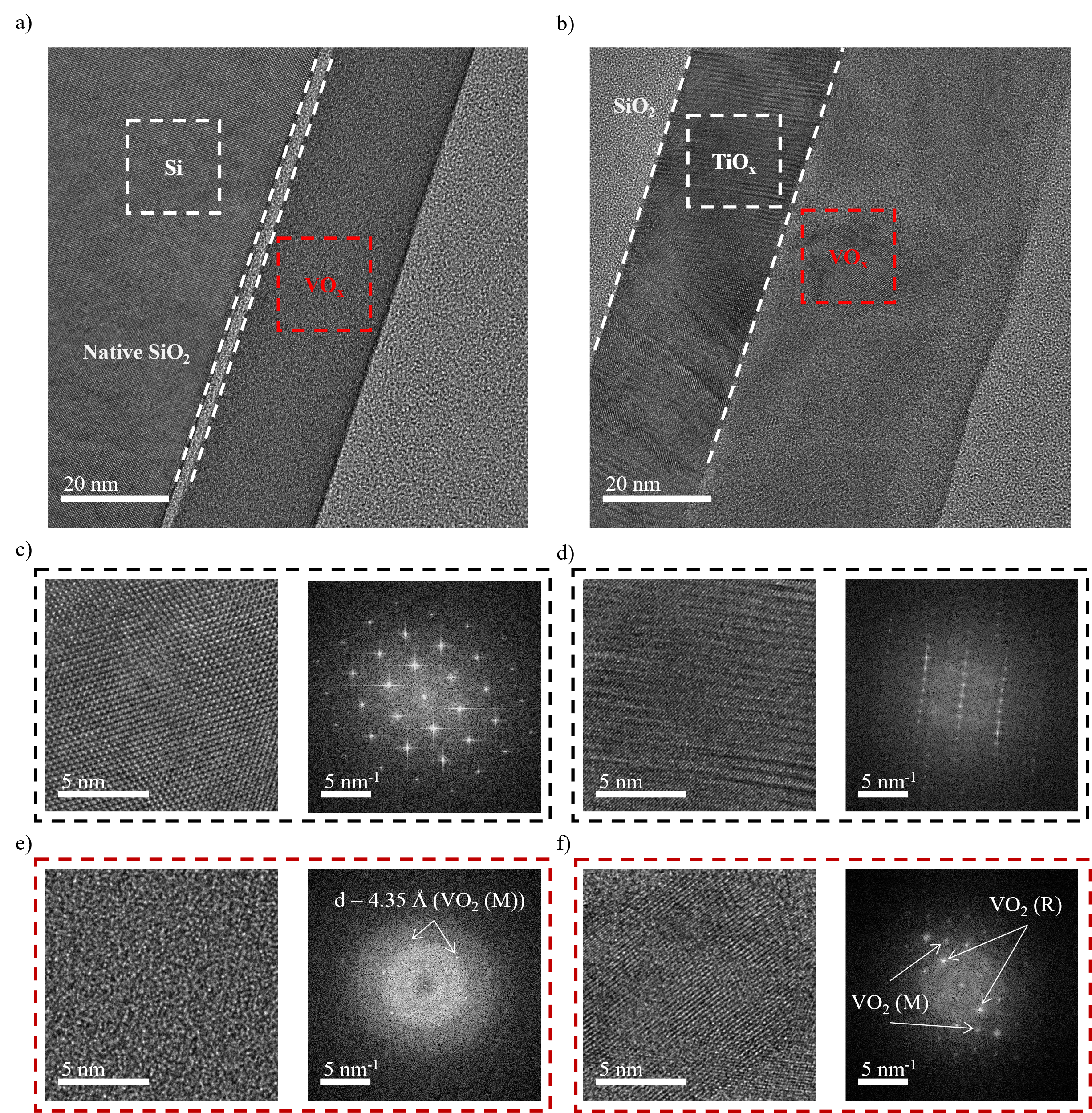


**Figure S7**. Cross-sectional HR-TEM images of VO_x_ deposited (a) on a Si wafer and (b) a TiO_x_-buffered Si wafer deposited at 300 °C. The fast fourier transformed (FFT) patterns of (c) Si wafer, (d) TiO_x_ buffer layer, (e) VO_x_ on Si wafer and (f) VO_x_ on TiO_x_-buffered Si wafer were obtained from HR-TEM images. (d) The stripes in TEM images and FFT patterns indicate that TiO_x_ buffer layer is crystallized during the VO_x_ deposition. Ring patterns with blurry monoclinic (010) VO_2_ spots in (e) indicate that the VO_x_ film directly grown on Si wafers is mostly amorphous. However, for VO_x_ films grown on TiO_x_-buffered Si wafers, the FFT interplanar patterns for rutile (111) VO_2_ and monoclinic (010) VO_2_ are clearly observed, implying its high crystallinity.

**Supplementary Note 7. CH W_x_V_1-x_O_y_** **multilayer without TiO_2_ separation layer**


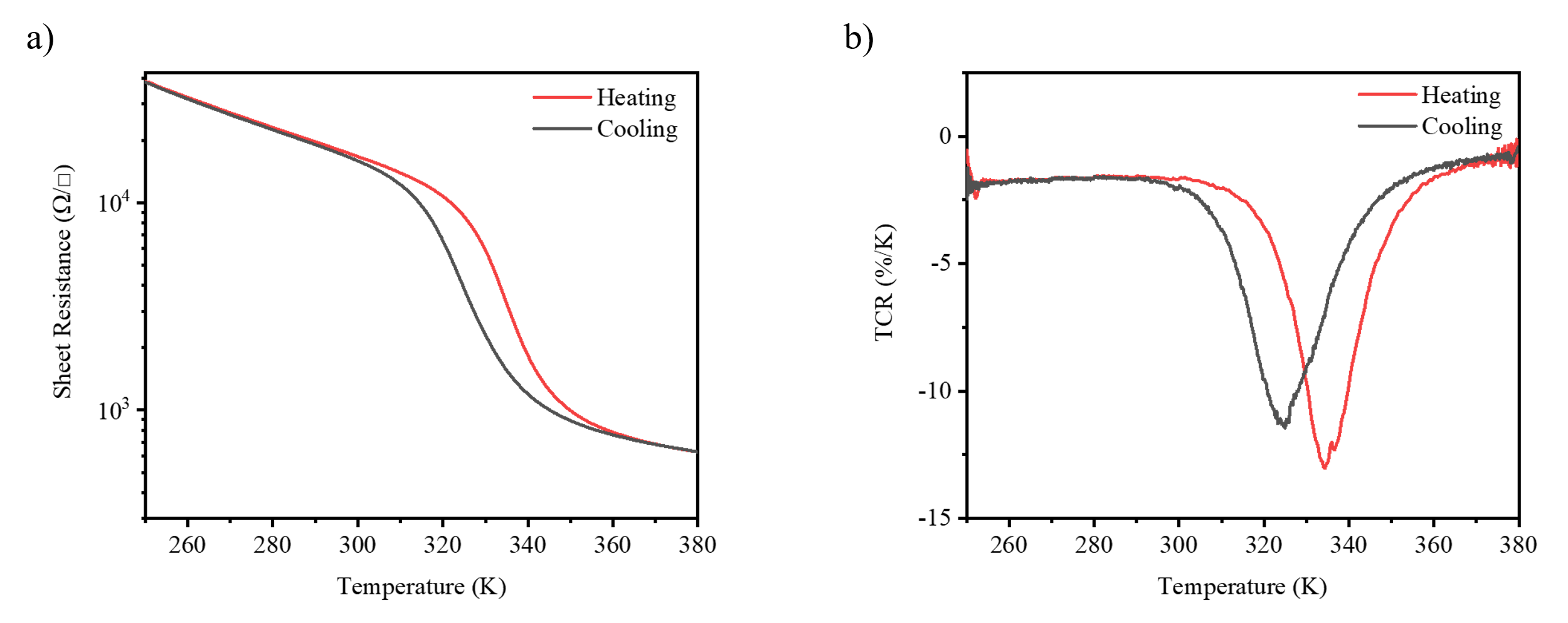


**Figure S8**. (a) Resistance *vs*. temperature curve of the CH W_x_V_1-x_O_y_ multilayer without separation layers and (b) the corresponding TCR curve obtained from the resistance *vs*. temperature data. The sharp peak of TCR curve indicates the intermixing of W dopant between W_x_V_1-x_O_y_ layers during deposition.

**Supplementary Note 8. The *I*–*V* curve and quadratic response of noise spectral densities of W_x_V_1-x_O_y_** **multilayers and TiO_x_ film**


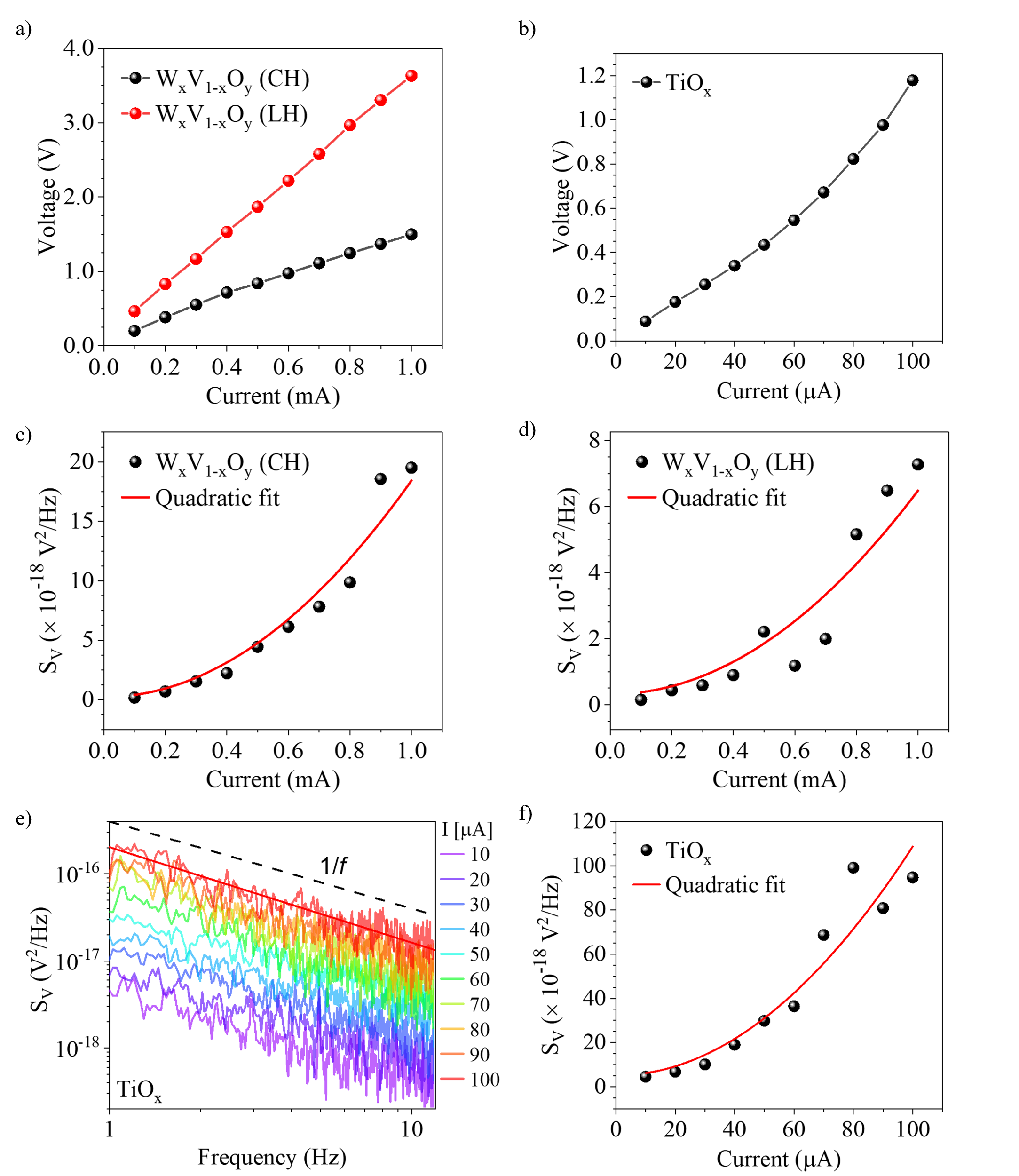


**Figure S9.** (a) Current–voltage (*I*–*V*) characteristics of the W_x_V_1-x_O_y_ multilayer and (b) TiOₓ thin film, both exhibiting nearly linear ohmic behavior. (c, d, f) Voltage noise spectral densities (*S_V_*) at 1 Hz under varying current biases for the CH and LH W_x_V_1-x_O_y_ multilayers and TiO_x_ film, respectively. (e) Full noise spectra of the TiO_x_ film under different current biases, with the black dashed line indicating a 1/*f* reference slope and the red solid line showing a 1/*f* fit at 100 μA. The *γ*/*n* values extracted from *S_V_* vs. current bias fitting (red curves) are 1.27 × 10^-30^ m^3^ for CH, 6.90 × 10^-32^ m^3^ for LH multilayers and 7.87 × 10^-30^ m^3^ for TiO_x_ film.

The noise spectral densities of TiO_x_ and W_x_V_1-x_O_y_ multilayers are measured under varying current biases. To ensure accurate noise measurements, all data were acquired within the ohmic regime, as confirmed by the linear *I*–*V* characteristics shown in Figures S9(a) and (b), which also indicate good electrical contact. To capture low-frequency behavior, the spectrum acquisition time was set to 32.79 s, enabling reliable data collection down to 0.0305 Hz (the inverse of the acquisition time). A low-pass filter with a cutoff at 30 Hz was applied to suppress high-frequency noise artifacts. As shown in Figure S9(e) and Figure 4(a, b), the spectra clearly follow 1/*f* noise behavior in the low-frequency region. The voltage noise spectral density (*S_V_*, in V^2^/Hz) at 1 Hz for the CH and LH W_x_V_1-x_O_y_ multilayers and TiO_x_ film are plotted in Figure S9(c), (d) and (f), respectively, under different current biases. All three samples display a clear quadratic dependence of *S_V_* on bias current, consistent with Hooge’s empirical relation. With fitting function, A·*I*^2^ + B, both *γ/n* related values and thermal (Johnson’s) noise is also obtained as A and the offset B in fitting function. The extracted Johnson’s noise levels are on the order of 10^-19^ V^2^/Hz for multilayers and 10^-18^ V^2^/Hz, which are consistent with the resistance values inferred from the *I*–*V* curve (~1.4 kΩ for CH, 3.5 kΩ for LH W_x_V_1-x_O_y_ multilayers and 11.7 kΩ for TiO_x_ thin film). For the TiOx film, the extracted *γ/n* (7.87 × 10^-30^ m^3^) is in good agreement with previously reported results (8.33 × 10^-30^ m^3^) ^[2]^, supporting the reliability of the measurements.

**Supplementary Note 8. Images of electric contact pads deposited on samples for noise spectrum measurements**


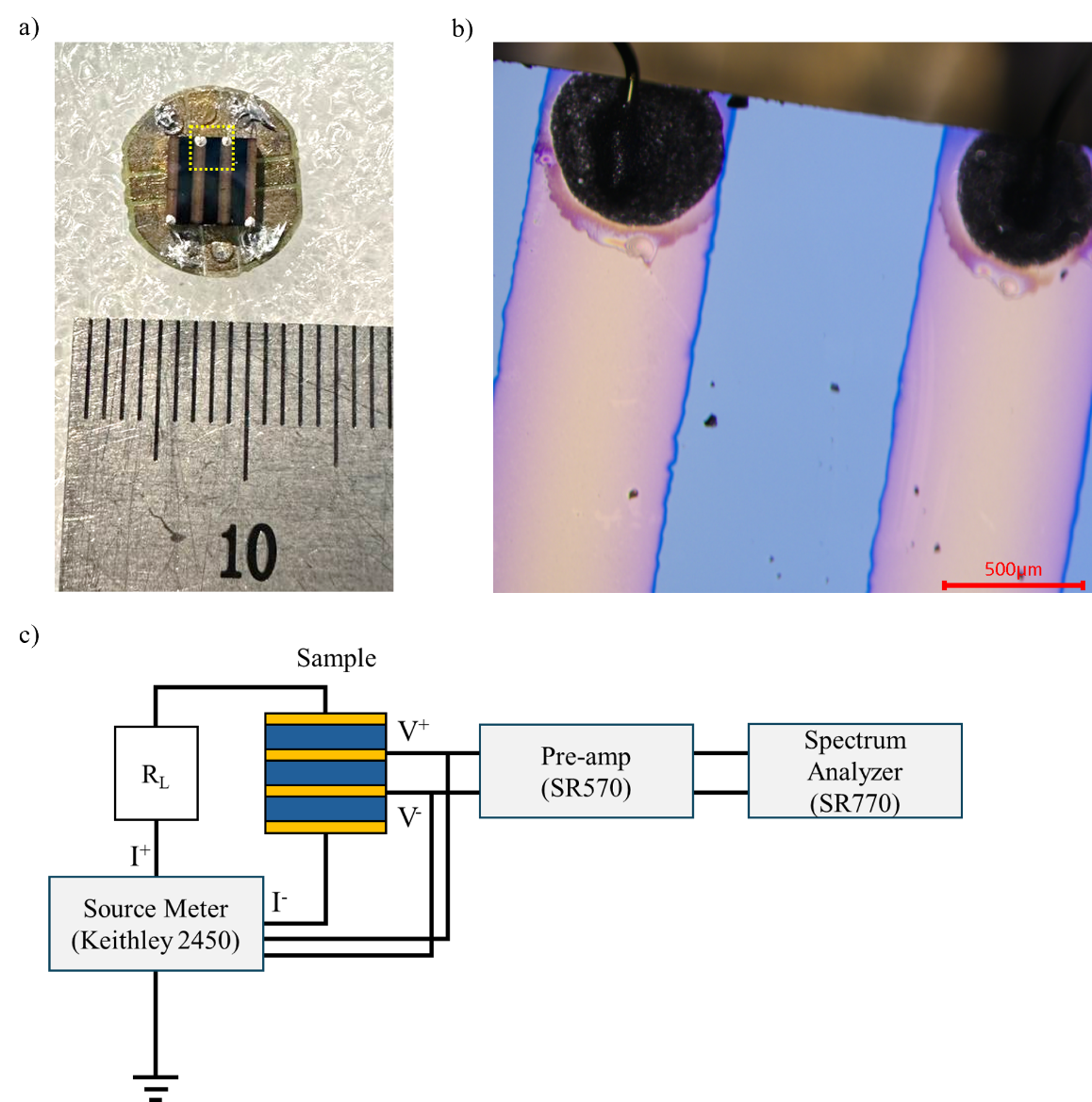


**Figure S10.** (a) The perks used for electric measurements. The samples are bonded with perks using GE varnish and Au wires are attached with perks by soldering indium. The yellow square corresponds to (b) the image obtained by optical microscope. Au electric contact pads deposited onto TiO_x_ film sample with Au wire bonded with Ag epoxy. (c) The schematic image of samples under noise spectra measurements. All samples are precisely diced to the size of 5 × 5 mm^2^ and the thicknesses measured with XRR are used to evaluate the volume of the samples (0.77 mm × 5 mm × thickness).

**Supplementary Note 9. Bolometric performances of previously reported materials**

|  | resistivity  [Ω cm] | TCR  [% K^-1^] | *γ*/*n*  [m^-3^] | *β* (\|TCR\|/√(*γ*/*n*))  [% K^-1^ m^-3/2^] | CMOS  compatibliliy | refs |
| --- | --- | --- | --- | --- | --- | --- |
| W_x_V_1-x_O_y_  CH multilayer | 2.71 × 10^-2^ | 4.5 | 1.27 × 10^-30^ | 4.02 × 10^15^ | O | This work |
| W_x_V_1-x_O_y_  LH multilayer | 4.90 × 10^-2^ | 4.5 | 6.90 × 10^-32^ | 1.72 × 10^16^ |  |  |
| W_x_V_1-x_O_2_ multilayer  (Experimental) | 0.1 – 0.012 | 10.5 | - | - | X | ^[3]^ |
| W_x_V_1-x_O_2_ multilayer  (Simulation) | 1.625 | 6.7 | - | - | O | ^[4]^ |
| α-VO_x_ | 0.67 | 2.1 | - | - | O | ^[5-7]^ |
|  | 0.13 | 1.87 | - | - |  |  |
|  | 0.02 | 1.25 | - | - |  |  |
|  | - | 1.70 | 2.7 × 10^-27^ | 3.27 × 10^13^ |  |  |
| α-VO_x_:W | 0.11 | 2.98 | - | - | O | ^[8]^ |
| α-VO_x_:Mo | 0.44 | 2.63 | - | - | O | ^[7]^ |
|  | 0.66  0.109 | 2.30  2.22 | -  - | -  - |  |  |
|  | 0.128 | 2.03 | - | - |  |  |
|  | 0.182 | 1.71 | - | - |  |  |
| TiO_x_ | - | 2.10 | 8.30 × 10^-30^ | 7.29 × 10^14^ | O | ^[2, 9]^ |
|  | 0.27 | 2.36 | 3.35 × 10^-28^ | 1.29 × 10^14^ |  |  |
|  | 0.19 | 2.25 | 1.59 × 10^-28^ | 1.78 × 10^14^ |  |  |
|  | 0.14 | 2.09 | 6.17 × 10^-29^ | 2.66 × 10^14^ |  |  |
| α-Si:Ge | 7.50 × 10^2^ | 1.80 | 10^-26^ | 1.8 × 10^13^ | O | ^[10-11]^ |
|  | 4.50 × 10^3^ | 6.50 | 10^-24^ | 6.5 × 10^12^ |  |  |
|  | 1.20 × 10^4^ | 2.80 | 10^-26^ | 2.8 × 10^13^ |  |  |
|  | 5.00 × 10^4^ | 5.00 | 10^-23^ | 1.58 × 10^12^ |  |  |
| α-Si:H | 2.50 × 10^2^ | 2.69 | 2.00 × 10^-26^ | 1.90 × 10^13^ | O | ^[10]^ |
| Ni_1-x_O | 9.04 | 1.61 | 1.27 × 10^-27^ | 4.52 × 10^13^ | O | ^[12]^ |
|  | 0.12 | 2.87 | 8.30 × 10^-27^ | 3.15 × 10^13^ |  |  |
| La_0.7_(Pb_0.63_Sr_0.37_)_0.3_MnO_3_ | 0.105 | 1.56 | 9.12 × 10^-28^ | 5.17× 10^13^ | X | ^[6]^ |
| La_0.67_Ca_0.33_MnO_3_ | 0.1 | - | 2× 10^-29^ | - | X | ^[13]^ |

**Table S1**. Bolometric parameters of different CMOS-compatible bolometric materials, including commercial amorphous VO_x_ (1.3 < x < 2.0). The resistivities and TCR values are used in Figure 3, while Hooge’s parameters (*γ*/*n*) and figures of merits (*β*, universal bolometric parameter) are used in Figure 4 ^[3-14]^. The CMOS-compatibility (low temperature synthesis and Si-based substrate compatibility) is denoted in the table.

The bolometric performance of commercial and previously reported materials is presented in Table S1. Note that the comparison plots shown in Figure 3 and Figure 4 include only near room temperature properties of CMOS-compatible materials; therefore, the W_x_V_1-x_O_2_ multilayer ^[3]^, La_0.7_(Pb_0.63_Sr_0.37_)_0.3_MnO_3_ ^[6]^, and La_0.67_Ca_0.33_MnO_3_ ^[13]^ are excluded. Our W_x_V_1-x_O_y_ multilayer in both CH and LH configurations exhibit a higher than the highest previously reported value (7.29 × 10^14^).

**References.**

[1] A. Glavic, M. Bjorck, "GenX 3: the latest generation of an established tool", *Journal of Applied Crystallography* 2022, **55**, 1063, https://doi.org/10.1107/S1600576722006653.

[2] M.-H. Kwon, K. Yang, Y.-S. Park, Y.-H. Kim, H. Chung, "Investigations of reactively sputtered TiO_2-delta_ films for microbolometer applications", in *Proceedings of the SPIE*, *Electro-Optical and Infrared Systems: Technology and Applications V*, vol. **7113** (2008): 711306, https://doi.org/10.1117/12.800023

[3] N. Émond, A. Hendaoui, M. Chaker, "Low resistivity W_x_V_1−x_O_2_-based multilayer structure with high temperature coefficient of resistance for microbolometer applications", *Applied Physics Letters* 2015, **107**, 143507, https://doi.org/10.1063/1.4932954.

[4] C. Wheeler, Y. Zhu, K. Sun*, et al.*, "Multilayer W-doped vanadium dioxide thermal sensors with extended operation region", *iScience* 2025, **28**, 112528, https://doi.org/10.1016/j.isci.2025.112528.

[5] P. Ashok, Y. S. Chauhan, A. Verma, "Low temperature synthesis of VO_2_ and hysteresis free VO_x_ thin films with high temperature coefficient of resistance for bolometer applications", *Thin Solid Films* 2023, **781**, 139975, https://doi.org/10.1016/j.tsf.2023.139975.

[6] A. Lisauskas, S. I. Khartsev, A. Grishin, "Tailoring the colossal magnetoresistivity: La_0.7_(Pb_0.63_Sr_0.37_)_0.3_MnO_3_ thin-film uncooled bolometer", *Applied Physics Letters* 2000, **77**, 756, https://doi.org/10.1063/1.127109.

[7] A. Ozcelik, O. Cabarcos, D. L. Allara, M. W. Horn, "Vanadium Oxide Thin Films Alloyed with Ti, Zr, Nb, and Mo for Uncooled Infrared Imaging Applications", *Journal of Electronic Materials* 2013, **42**, 901, https://doi.org/10.1007/s11664-012-2326-9.

[8] O. Celik, M. Duman, "High temperature coefficient of resistance and low noise tungsten oxide doped amorphous vanadium oxide thin films for microbolometer applications", *Thin Solid Films* 2019, **691**, 137590, https://doi.org/10.1016/j.tsf.2019.137590.

[9] Q. Zhang, R. Yan, X. Peng, Y. Wang, S. Feng, "TiO_2−x_ films for bolometer applications: recent progress and perspectives", *Materials Research Express* 2022, **9**, 012002, https://doi.org/10.1088/2053-1591/ac4327.

[10] V. Y. Zerov, V. G. Malyarov, "Heat-sensitive materials for uncooled microbolometer arrays", *Journal of Optical Technology* 2001, **68**, 939, https://doi.org/10.1364/JOT.68.000939.

[11] S. Ajmera, A. Syllaios, G. Tyber, M. Taylor, R. Hollingsworth, "Amorphous silicon thin-films for uncooled infrared microbolometer sensors", in *Infrared Technology and Applications XXXVI*, *SPIE Defense, Security, and Sensing*, vol. **7660** (2010): 766012, https://doi.org/10.1117/12.850545

[12] I.-K. Kang, Y. A. K. Reddy, Y. B. Shin, W. Y. Kim, H. C. Lee, "Enhanced bolometric properties of nickel oxide thin films for infrared image sensor applications by substitutional incorporation of Li", *Ceramics International* 2018, **44**, 7808, https://doi.org/10.1016/j.ceramint.2018.01.213.

[13] P. Reutler, A. Bensaid, F. Herbstritt*, et al.*, "Local magnetic order in manganite thin films studied by 1/*f* noise measurements", *Physical Review B* 2000, **62**, 11619, https://doi.org/10.1103/PhysRevB.62.11619.

[14] B. Rajeswaran, A. M. Umarji, "Effect of W addition on the electrical switching of VO_2_ thin films", *AIP Advances* 2016, **6**, 035215, https://doi.org/10.1063/1.4944855.
